# A Review of the Quadratus Lumborum Block and ERAS

**DOI:** 10.3389/fmed.2018.00044

**Published:** 2018-02-26

**Authors:** Michael Akerman, Nada Pejčić, Ivan Veličković

**Affiliations:** ^1^Weill Cornell Medical College, Cornell University, New York City, NY, United States; ^2^General Hospital Leskovac, Leskovac, Serbia; ^3^SUNY Downstate Medical Center, Brooklyn, NY, United States

**Keywords:** quadratus lumborum, truncal block, quadratus lumborum block, transversus abdominis plane block, ultrasound

## Abstract

The use of truncal nerve blocks has been described since 2001. Since then, there have been many studies trying to understand the ideal clinical scenarios for its use. Since 2001, the transversus abdominis plane block has evolved in many ways including from landmark based technique to ultrasound guided and more recently, into the quadratus lumborum (QL) block. Its anatomical placement, concentration of local anesthetic, volume of local anesthetic, and anatomic placement have all been raised as clinical questions. This article will discuss the literature of the QL block in an effort to understand how it is best used in a variety of clinical scenarios.

## Introduction

The truncal nerve blocks, as a part of perioperative pain management, were introduced into clinical practice over 40 years ago. Primarily these were the ilioinguinal–iliohypogastric (II–IH) block (1–4) and the rectus sheath block (5), mostly used in the pediatric anesthesia population. In the early years of the 21st century, the transversus abdominis plane (TAP) block was introduced in everyday practice, providing a much wider field of analgesia (6, 7). At first, these blocks were performed without ultrasound guidance, using landmark techniques. However, the clinical use of truncal block techniques have developed over time and their expansion was driven by introducing ultrasound into anesthesiology practice. Although the anatomical markers are reliably detected by ultrasound, the blocks of the anterior abdominal wall vary in both the distribution of the local anesthetics and the field of coverage. In the search for the wider analgesia coverage and long-lasting postoperative analgesia, the transversalis fascia plane block and the quadratus lumborum block (QLB) have been developed.

Quadratus lumborum block is a block of the posterior abdominal wall, “interfascial plane block,” which is performed exclusively under ultrasound guidance. It was described by anesthesiologist Dr. Rafael Blanco (8) as a variant of the TAP block in 2007. Much later, he gave a detailed description of the block technique using the name QLB (9). In the spring of 2013, Dr. Jens Børglum from the University Hospital in Copenhagen (Denmark) published a new ultrasound-guided transmuscular QL blockade, describing the so-called “Shamrock sign,” the sign of a shamrock for the detection of a local anesthetic injection point (10). In autumn 2013, Dr. MihaelaVisoiu (11), a pediatric anesthesiologist from the University Children’s Hospital in Pittsburgh (USA), published a case report with continuous QLB for postoperative analgesia. Subsequently, there has been an increasing interest of the anesthesia community in the use of truncal blocks, and the number of publications on the topic of QLB is progressively growing.

## Mechanism of Action

The crucial ultrasound landmark for block performance is the quadratus lumborum muscle (QLM), and the key to the analgesia lies in the thoracolumbar fascia (TLF) (12–15). TLF is a complex, connective tissue tubular structure formed by binding aponeuroses and fascia layers, which, enveloping the back muscles, connects the anterolateral abdominal wall with the lumbar paravertebral region. The TLF is on its medial side attached to the thoracic and lumbar vertebrae, cranially continuing with endothoracic, and caudally with the fascia iliaca, potentially ensuring the spread of anesthetics in the craniocaudal direction (16). The true mechanism of analgesia provided by QLB has not yet been fully clarified. It is believed that the local anesthetics spread along the TLF and the endothoracic fascia into the paravertebral space, is responsible in part for the analgesia. In 2011, Carney et al. (17) showed that contrast spreads from the L1–T5 segment of the paravertebral space. However, a recent publication (18), shows that contrast injected into the area around QLM (QL plane) does not spread into the paravertebral space and contrast injected into the paravertebral space does not spread around QLM. Hence the assumption that visceral analgesia results from the spread of anesthetics to the celiac ganglion or sympathetic trunk *via* splanchnic nerves, as is the case with the paravertebral block. This remains to be confirmed or denied by future research. The most recent publication on this topic is the abstract presented at the American Society of Anesthesiologists meeting in October 2017, which shows local anesthetic spreading into the paravertebral space, cranially to the T10 segment (19).

An additional mechanism of action of local anesthetics can be explained by the anatomical–histological characteristics of the TLF. Namely, in the superficial layer of the TLF, there is a thick network of sympathetic neurons. In the fascia, there are the high-threshold and low-threshold mechanoreceptors and pain receptors sensitive to the effects of the local anesthetics. These receptors play a role in the development of both acute and chronic pain. The QLB analgesia could be, at least partially, explained by local anesthetic blockade of these receptors (15, 20).

Different approaches to block performance are applied in everyday clinical practice, and differences in the width of the anesthetized field and the duration of analgesia are significant. So far, studies done on cadavers (18, 21–24) show that the injected contrast can spread cranially to the thoracic paravertebral space and intercostal spaces covering somatic nerves and the thoracic sympathetic trunk up to the T4 level. Blockade of the subcostal, iliohypogastric, and ilioinguinal nerve is consistent. Sometimes, genitofemoral and lateral femoral cutaneous nerve could be blocked. Caudally, contrast can reach lumbar nerve roots, but the results vary and new studies are needed to clarify the link between the type of QLB and the achieved analgesic effect. All of these data indicate that the QLB provides somatic and visceral analgesia.

Obviously, there are variations in the width of achieved analgesia, and in the number of dermatomes covered by QLB. In most of the cases, analgesia is achieved in T7–L1 dermatomes (10–14, 24–28), although there are descriptions of cranial spread to T4–T5 (13), and caudal spread to L2–L3 (22) dermatomes. The height of the block can be influenced by the choice of the site for the application of local anesthetics, both in relation to QLM and in relation to the distance from the iliac crest and costal margin (12, 13). The rate of the drug application (29), and the individual anatomical variations can also influence the height of the block.

## Types of QLB

Since the initial description, the block has experienced several modifications and today four types of the block are performed, which differ by the site of drug application. These are QLB 1 or lateral QLB, QLB 2 or posterior QLB, QLB 3, or anterior/transmuscular QLB, and QLB 4 or intramuscular QLB.

Quadratus lumborum block 1 implies the application of local anesthetics on the lateral side of QLM in the area of its contact with the transversalis fascia, at the level where transversus abdominis muscle (TAM) tapers off into its aponeurosis (30). One group of authors (12) states that the target site is between the fascia and the muscle, which can be seen as expanding space upon local anesthetic injection. They emphasize that medication should not be given between the fascial layers as the nerve endings are between the fascia and the muscle. Another group of authors (13) states that drug is administered in the space between the common aponeurosis of internal oblique muscle (IOM) and TAM and the transversalis fascia.

Quadratus lumborum block 2 implies the application of medication on the posterior side of the QLM between the QLM and the medial lamina of TLF which separates QLM from the latissimus dorsi muscle and paraspinal muscles [erector spinae muscles (ESM)]. This is laterally from the attachment of IOM aponeurosis (30), in the to the so-called lumbar interfascial triangle (14).

Quadratus lumborum block 3 implies the application of medication at the front of the QLM, at the level of its attachment to the transverse process of L4 vertebra. This can be seen under ultrasound as spreading of the local anesthetic between the QLM and the psoas major muscle (PMM) (10, 31). This approach assumes during ultrasound one is viewing the “Shamrock sign”—the transverse process of L4 vertebra is seen as a stem with ESM as posterior leaf, PMM as anterior leaf, and QLM as lateral leaf.

Quadratus lumborum block 4 implies the application of medication in the muscle itself.

Murouchi states that for QLB 1 and 3 a local anesthetic needs to be applied between the anterior layers of TLF and that its intramuscular approach does not involve the spread of local anesthetics into the interfascial space (28).

## Block Technique

**Figure 1:**
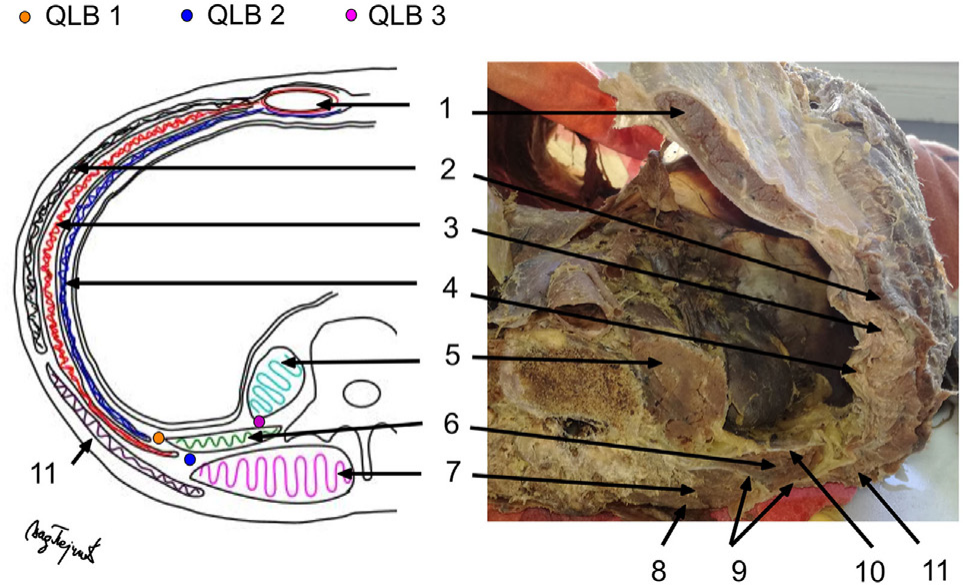
Cross-section of the abdomen—a photo of cadaver and a scheme of anatomical structures. QLB 1—point of local anesthetic (LA) injection for QLB 1; QLB 2—point of LA injection for QLB 2; QLB 3—point of LA injection for QLB 3; 1—rectus abdominis muscle; 2—external oblique muscle; 3—internal oblique muscle; 4 –transversus abdominis muscle; 5—psoas major muscle; 6—quadratus lumborum muscle; 7—erectores spinae muscle; 8—lamina posterior of the thoracolumbar fascia; 9—lamina media of the thoracolumbar fascia; 10—lamina anterior of the thoracolumbar fascia; 11—latissimus dorsi muscle.

**Figure 2:**
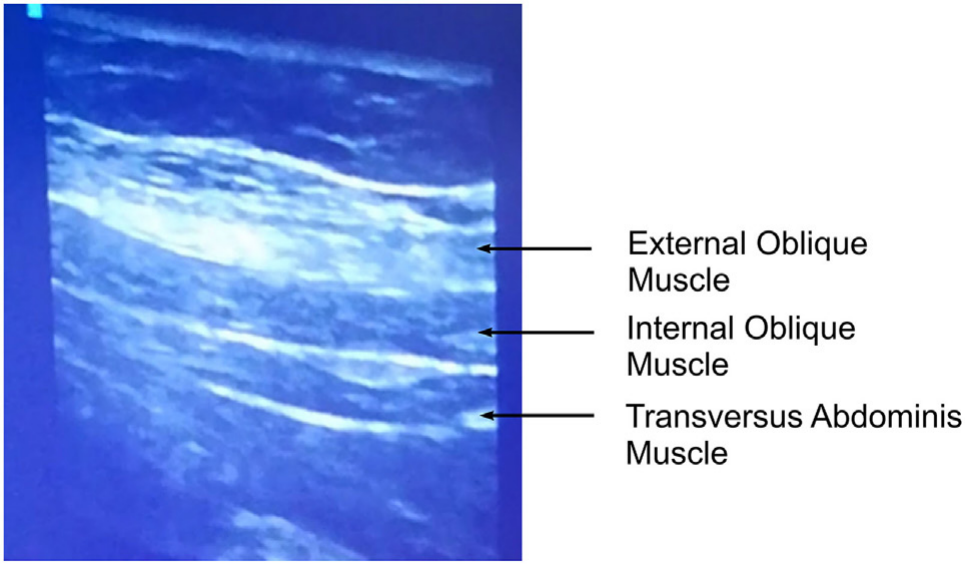
Muscles of the anterolateral abdominal wall.

**Figure 3:**
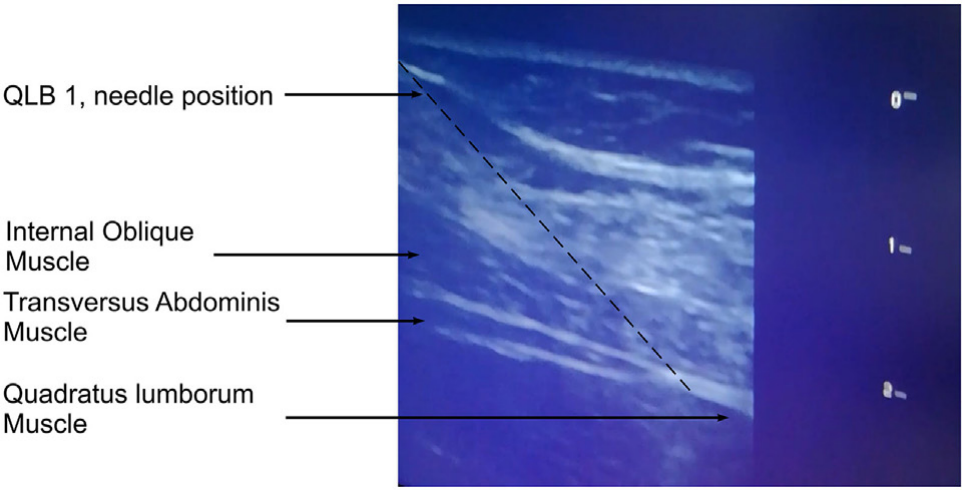
Quadratus lumborum type 1 block—needle position.

We perform lateral QLB (QLB 1) and posterior QLB (QLB 2) in our practice. During the block performance, the patient is in the supine position. Figure 1 shows the cross-section of the cadaver abdomen in supination and a schematic presentation of anatomical structures for better understanding and easier performance of the QLB. If QLB is performed on the operating table, the operating table can be gently tilted to the opposite side to achieve a better exposure. If QLB is performed on a regular bed, a pillow can be placed under the lumbar spine. Alternatively, the patient could be asked to turn to the opposite side. The procedure begins by placing a transversally oriented linear or convex ultrasound probe between two distinct markers—the iliac crest and the costal margin at the level of the anterior axillary line. The goal is to find three thin parallel muscles of the anterolateral abdominal wall, external oblique muscle, IOM, and TAM, from the outside to inside as in Figure 2. Moving the probe posteriorly, we follow narrowing of the muscles until the muscle fibers of TAM taper off into its aponeurosis at the level of the posterior axillary line. This is ultrasound-detected as a hyperechogenic sign (Figure 3), from which the QLM extends posteriorly and to the inside. Aponeuroses are seen as hyperechogenic structures, and muscles as hypoechogenic structures. If the image is lost during probe movement, we reposition probe to the starting point of scanning, looking for three parallel muscles, and then we continue scanning to the back, taking care that the probe is always perpendicularly placed on the skin surface and is following the body curvature. When we detect a remarkable hyperechogenic sign of the place where we want to inject a local anesthetic, we can improve the image by discrete tilting and rotation of the probe. If the hypoechogenic shadow blurs the image, it is necessary to add more gel that will improve the transmission of ultrasound waves from the probe to the skin.

The needle is introduced into the skin 1–2 cm above the probe (by “in plane” technique) and led through the muscles to the local anesthetic application site—posteriorly from the place where the TAM tapers off into its aponeurosis (we do not want the TAM perforation). The needle is advanced at 90° angle and only after the skin perforation we redirect needle in the desired direction. The TLF provides a characteristic elastic resistance to a blunt needle that is used for peripheral blocks, and TLF perforation is accompanied by double control—visual (ultrasound) and special tactile feeling due to loss of resistance. After a negative aspiration test, the injection of 1 ml of the solution provides visible hydrodisection—the accumulation of fluid in the form of a growing hypoechogenic shadow, which separates the muscle from the fascia, representing the third confirmation of the desired location. Then, the fractional administration of local anesthetics is performed. After every 5 ml of local anesthetic, it is necessary to do an aspiration test to confirm the extravascular location of the needle tip.

This technique is very easy to learn due to the fact that it is easy to find the key sonoanatomic markers for block performance. It can be learnt after only a few performance of the procedure (32).

When the catheter is placed for prolonged postoperative pain therapy, absolutely sterile working conditions are necessary (as for the epidural catheter placement or the central venous cannulation). This involves using caps, masks, sterile gloves, hand cleaning, sterile operating field, putting the probe into a sterile bag, and using sterile ultrasound gel. In the absence of sterile bags for the ultrasound probe and sterile gel, it is possible to improvise in the following way. An ultrasound probe with regular gel is placed in a sterile glove. The contact of the probe with skin can be improved by wetting skin with sterile saline.

For one-time administration of local anesthetic (“single shot” QLB), it is sufficient to apply clean technique according to many US regional anesthetic schools. This involves the use of regular gloves for single use without the preparation of a wide sterile field. It is necessary to use a cap and a mask. After identification of TAM and TLF, skin above the probe is cleaned with disinfectant. Insulated needle is held only at the “head,” (without touching the metal part of the needle), and advanced through the cleaned skin. Only when we have an adequate ultrasound image and disinfected skin, we advance the needle through the skin and perform the block. A group of authors (33) recommends that a sterile transparent film (Tegaderm) should be placed over the probe and gel to additionally increase the safety and reduce the risk of infection.

For the performance of QLB, insulated block needles 50–150 mm in length are used, although for most patients the appropriate needle length is 100 mm.

There is still no consensus on the type, concentration, and volume of a local anesthetic used to perform QLB. QLB is performed by applying 15–30 mL (0.2–0.4 ml/kg) of a local anesthetic on the left and right side of the abdominal wall. 0.125–0.375% bupivacaine, levobupivacaine, or ropivacaine (12–14, 23, 25, 30, 34–37) can be used as local anesthetics. Many authors (26, 31, 35) recommend the addition of 2–4 mg dexamethasone to each side to extend the effect of the local anesthetic and, by some authors (38, 39), achieve the antiemetic effect. There is still no consensus on the effect of dexamethasone on the duration of peripheral nerve blocks either, but the most recent meta-analyses (39–41) indicate that perineurally administered dexamethasone prolongs the duration of the peripheral block and potentiates analgesia. We are currently complying with the recommended protocol from the Cornell Medical Center (Weill Cornell Medicine, New York, NY, USA), taking care that the patient does not receive a local anesthetic dose higher than the maximum allowed (2.5 mg/kg). We use 30 ml 0.25% bupivacaine/levobupivacaine with 2–4 mg dexamethasone per block. As a bilateral QLB is required for most procedures, the total dose is 60 ml of 0.25% (150 mg) bupivacaine/levobupivacaine with 4–8 mg of dexamethasone. For patients with a body weight of less than 60 kg, we use 20–30 ml of 0.20% bupivacaine/levobupivacaine with 2 mg dexamethasone per side.

The block can be performed postoperatively, on the operating table, immediately after waking up patient from general anesthesia, in the recovery room or in the intensive care unit. Patients who underwent neuraxial anesthesia are given QLB either before or after resolution of the block.

## Indications

Quadratus lumborum block provides postoperative analgesia in a large number of surgical interventions and the list of indications is long.

The efficacy of QLB for postoperative analgesia following both cesarean section (8, 14, 30, 34, 35, 42–44) and gynecological laparoscopic procedures (25, 36) was shown. Additionally, the efficacy of QLB for postoperative analgesia was shown after abdominal surgery [small intestine (26) and colon (11, 27) resection, colostomy reconstruction (11), appendectomy (27), gastrectomy (45)], and for analgesia for anterior abdominal wall hernioplasty (46, 47) and orhcydopexy (47), both for open and laparoscopic procedures and for postoperative analgesia after open and laparoscopic nephrectomy (37, 48, 49).

As TAP block has its important place in postoperative analgesia after laparoscopic cholecystectomy, Elsharkawy (50) representing the American Society of Regional Anesthesia and Pain Medicine recommends the application of QLB for laparoscopic cholecystectomy (which we have confirmed in everyday practice on over 50 patients for the past 6 months).

There are more and more authors describing the application QLB for hip and femur surgery (31, 51–55) and lumbar vertebrae surgery (56, 57). A case study of the use of QLB for postoperative analgesia after femorofemoral bypass was published (58). A case of one-time administration of QLB in chronic pain treatment after the anterior abdominal wall hernia surgery with a multimonth effect after the block performance was also published (46).

## QLB Efficacy

All authors, which we have quoted so far, agree that QLB has an outstanding analgesic effect on pain reduction to 1–2/10 by Visual Analog Scale or Numeric Rating Scale pain scale, which usually last more than 24 h. Patients who receive QLB as part of a postoperative pain therapy, have lower pain levels both when resting and moving, which is important for early mobilization. The analgesic effect is as good as the one achieved by opioids, and there are no unwanted opioid effects such as nausea and vomiting (36). According to prospective studies published by Blanco et al.1 (4, 30) in 2015 and 2016, the need for morphine has been significantly reduced postoperatively in patients who received paracetamol, NSAID, and QLB as part of the multimodal postoperative analgesia compared to patients who received only paracetamol and NSAID, but did not receive QLB. Comparative studies have shown that the QLB covers a topographically broader field (Th7–Th12, compared to TAP Th10–Th12) (14, 25), and yields prolonged pain-free condition compared to the TAP block (24–48 h QLB versus 8–12 h TAP block) (14, 25, 47).

Quadratus lumborum block provides early and rapid pain relief in a high percentage of patients and allows early ambulation, which is one of the most important measures in the prevention of deep vein thrombosis and thromboembolic complications. So, this would be another important question that should be considered through future research—could QLB be used to reduce the incidence of postoperative thromboembolic complications?

## QLB Complications

Complications associated with the performance of abdominal wall blocks are fortunately very rare and not described during QLB performance. Since QLB is a classical intramuscular medication injection, the possibility of infection is far lower than in performing the neuraxial blocks. So far, infections have not been described during the QLB performance. The advantage of QLB compared to other abdominal wall blocks is the fact that the passage of the needle and the site of the local anesthetic application are very distant from the peritoneal cavity, visceral abdominal organs, and large blood vessels. Therefore, needle trauma in terms of unintentional puncture of the peritoneum, intestine, liver, kidney, large blood vessels associated with blind methods (without ultrasound) of the TAP and II-IH block performance here is minimized. Performing a block under the control of ultrasound, with mandatory monitoring of the needle tip prior to injecting the drug, significantly increases the level of safety and efficiency of the technique. There are no data on neurological damage since the local anesthetic is not injected into the immediate proximity of the large nerve, but is injected into the space rich in small nerve endings. It is therefore generally accepted that QLB can be performed both under general and regional anesthesia (13).

An unwanted femoral nerve block is cited as a possible complication of QLB 3. A rational theoretical explanation lies in the immediate anatomical contact of the TLF and the iliac fascia and the possibility of spreading the anesthetic, down the iliac fascia causing weakness in the quadriceps (22, 52, 53, 59). Dam and associates (21) during the performance of QLB 3 do not puncture the PMM and do not get the contrast spreading caudally. This leaves us with a potential conclusion that if there are no punctures of the PMM, there is no unwanted quadriceps weakness.

Anterior abdominal wall blocks have the potential for local anesthetic systemic toxicity (LAST). For now, there is no LAST case with QLB. Namely, studies have shown that the concentration of local anesthetic (ropivacaine) in plasma is significantly lower after the performance of QLB comparing with than in the TAP block done by a lateral approach (13, 25). In any case, whenever regional blocks are performed, it is necessary to think of a potential LAST, take precautions to prevent LAST development and actively monitor the patient to timely spot the first signs and treat LAST.

As the QLB performance involves manipulation of the fascia where blood vessels exit from the paravertebral space, caution should be exercised in people receiving anticoagulant therapy due to the possible risk of hematoma (14).

As with any anesthetic procedure, it is necessary to take patient’s written consent for the performance of the abdominal wall block, especially if the block is performed postoperatively in the intensive care unit or ward (13).

## QLB and ERAS Protocol

Our review of literature did not result in any articles that would specifically discuss the role of QLB in ERAS protocols. Kim et al. recently published review on the role of TAP block as a part of ERAS protocol (60). They found that the use of TAP block resulted in significantly less opioid use, less postoperative pain and non inferiority was shown in comparison with thoracic epidural. Since QLB is similar to TAP block all of these findings should be subjects of new research. Other studies have shown less post-operative nausea and vomiting (36, 61), decreased post-operative sedation (62, 63), decreased length of hospital stay (64), earlier urinary catheter removal (65) when abdominal trunk blocks are used. This is another area where extensive research is needed. Improved early oral intake and early mobilization can be more easily achieved with good pain control and QLB has a great potential in this area of ERAS.

## Conclusion

Quadratus lumborum block is a new form of the abdominal wall block which is relatively easily performed thanks to clear ultrasound anatomic markers. The block effect lasts 24–48 h and until now no complications have been described during the block performance. QLB is safe and has found its place in multimodal postoperative pain therapy in patients undergoing abdominal surgery, gynecological and obstetric procedures, and orthopedic interventions on hips, whether interventions are performed in general or spinal anesthesia, both in adults and in children. It follows from the above that QLB has the potential to significantly facilitate and improve postoperative pain therapy.

## Author Contributions

MA performed the proofreading of the translation of the article. NP and IV did the writing in Serbian and the literature search for the article.

## Conflict of Interest Statement

The authors declare that the research was conducted in the absence of any commercial or financial relationships that could be construed as a potential conflict of interest. The reviewer DZM and the handling Editor declared their shared affiliation.
